# Supplementary material for: Melanistic coloration does not influence thermoregulation in the crepuscular gecko Eublepharis macularius
Source: Biol Open. 2023 Nov 10;12(10):bio060114. doi: 10.1242/bio.060114 (PMC10651090; doi:10.1242/bio.060114)
Supplement: Supplementary information [file biolopen-12-060114-s1.pdf]

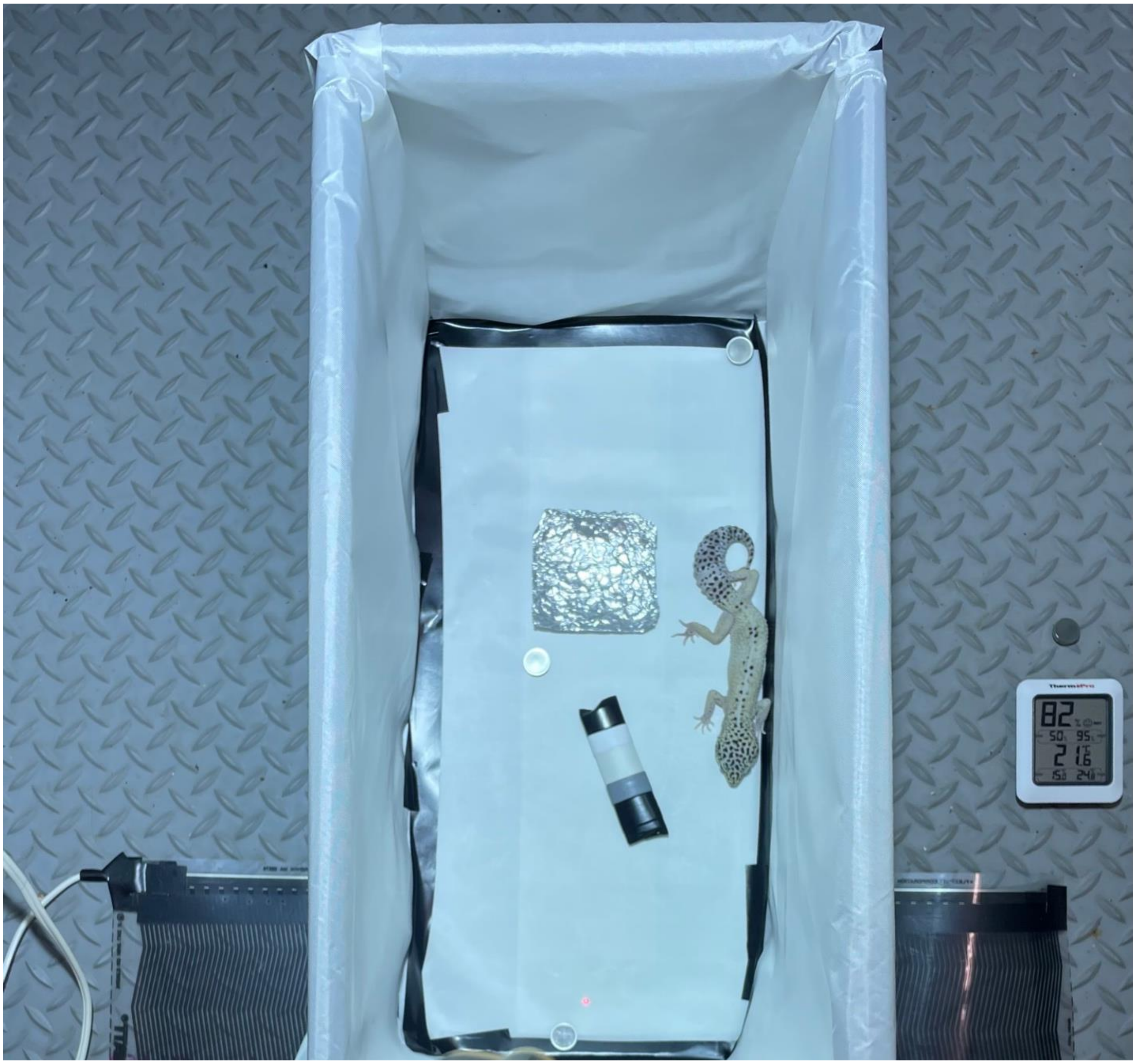

Fig. S1. Experimental setup. Testing terrarium covered in white sheets of Teflon bordered by black electrical tape. A heating pad is set up at the warm end of the terrarium. Three iButton dataloggers are inside on the opposite ends and in the middle (where the cardboard hide would be while not capturing images), while one is outside with a digital thermometer/hygrometer. The standards used for image capture are temporarily positioned in the terrarium in range of the gecko.

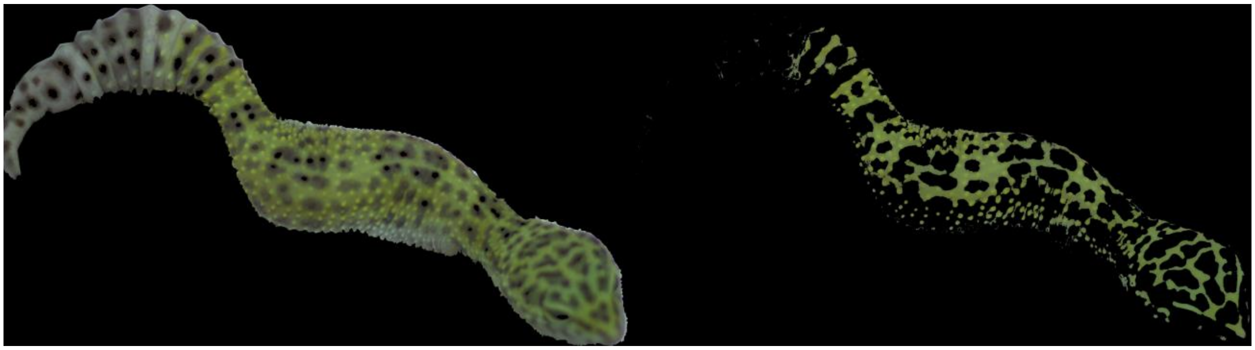

Fig. S2. Difference in *k* selection for clustering algorithm. A conservative clustering of *k*=2 (left) was used as to not overestimate the melanistic proportion present in an individual, which occurred at *k*>2 (right).

Table S1. Spearman correlation coefficients between body temperatures obtained from different parts of the body. Body temperature was taken from the Infrared images using the average of the two eyes and tested for correlation of temperatures of other body regions.

**Body region where the temperature was obtained from**	**Spearman correlation**	**P-values**
Snout	0.991	p<0.001
Head	0.989	p<0.001
Dorsum	0.985	p<0.001
Back Leg	0.988	p<0.001
Front Foot	0.985	p<0.001
Tail	0.984	p<0.001

Biology Open • Supplementary information
